# Fast Synthesis of Pt Nanocrystals and Pt/Microporous La_2_O_3_ Materials Using Acoustic Levitation

**DOI:** 10.1186/s11671-018-2467-8

**Published:** 2018-02-13

**Authors:** Yinkai Yu, Shaohua Qu, Duyang Zang, Liuding Wang, Hongjing Wu

**Affiliations:** Department of Applied Physics, Key Laboratory of Space Applied Physics and Chemistry of Ministry of Education, School of Sciences, Northwestern Polytechnical University, Xi’an, People’s Republic of China

**Keywords:** Single-atom Pt, Pt/microporous La_2_O_3_, Acoustic levitation

## Abstract

Usually, we must use an appropriate support material to keep the metal species stable and finely dispersed as supported metal nanoparticles for industry application. Therefore, the choice of support material is a key factor in determining the dispersion and particle size of the noble metal species. Here, we report the synthesis of a single-atom Pt material in the solution and supported Pt nanoclusters on microporous La_2_O_3_ by a one-step acoustic levitation method without any pretreatment/modification of raw oxide. We have strongly contributed to the synthetic methodology of the surface/interfacial heterogeneous catalysts in this study, and this finding could open another door for synthesis of supported metal nanoparticles on porous materials for environmental catalysis.

**Electronic supplementary material:**

The online version of this article (10.1186/s11671-018-2467-8) contains supplementary material, which is available to authorized users.

## Background

Since the first practical isolated single-atom Pt on FeO_*x*_ catalyst was realized by Qiao et al. [1], the concept of “single-atom catalysis” has attracted increasing research attention. Downsizing Pt nanoparticles to clusters or even single atom could largely improve the catalytic activity and is therefore able to increase the active surface area of the catalyst. However, the large-scale synthesis of practical and stable Pt cluster and single atoms of catalysts remains a significant challenge because clusters and single atoms possess too high surface free energy and are easy to sinter under realistic reaction conditions [2, 3].

During the past decade, there are only a few strategies to atomically disperse metal sites on catalyst supports. For example, defects on reducible oxides help to stabilize atomically dispersed metal atoms on supports in the form of metal-O-support bonding [4]. Coordinatively unsaturated Al^3+^ ions on Al_2_O_3_ support act as binding centers to maintain the high dispersion of Pt atoms, but the loading amount of metal components must be low [5]. One major challenge remains in the field of atomically dispersed catalysts: to choose the optimal supports for atomically dispersed metal atoms. Recently, Li et al. [6] reported that a deposition process was developed to fabricate a single-atom layer Pt coating on a complicated 3D (three-dimensional) Ni foam substrate using a buffer layer (Au or Ag) strategy. The Pt monolayer is found to work as well as a thick Pt film for catalytic reactions [7].

Here, we proposed an acoustic levitation method to prepare monodispersed Pt nanoclusters and even single-atom Pt in the solution. Though the single-atom Pt is a small part of the overall Pt morphology, we have also contributed to the synthetic methodology of the single-atom Pt material in the solution. Furthermore, Pt/microporous La_2_O_3_ could be one-step prepared by the acoustic levitation method without any pretreatment/modification of raw oxide. Based on the XPS (X-ray photoelectron spectroscopy) analysis, we can infer that the La_2_O_3_ oxide layer indeed covers and contacts with the Pt metal, leading to the formation of surface La–O–Pt species, on which abundant oxygen defects can be created to facilitate the electrophilic oxidation reaction.

## Results and Discussion

**Fig. 1:**
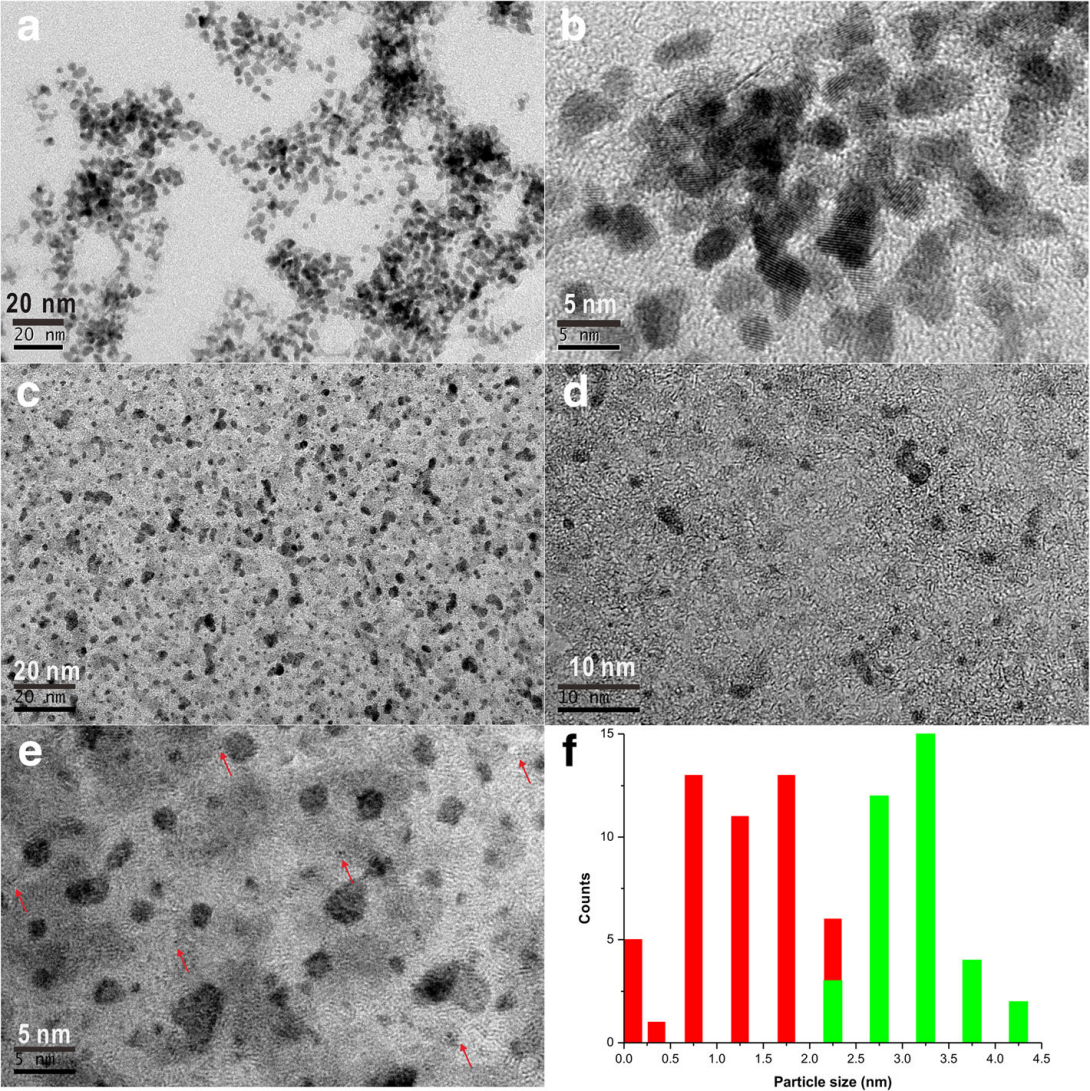
HRTEM images of **a**, **b** Pt nanoparticles prepared by the reduction by NaBH_4_ without acoustic levitation (0.5 g/L), **c–e** Pt clusters prepared by the acoustic levitation (0.5 g/L), and **f** particle size distributions for the above two Pt colloids (green for NaBH_4_ reduction, red for acoustic levitation)

High-resolution transmission electron microscopy (HRTEM) was used to characterize the dispersion and configuration of the Pt cluster in the samples. Figure 1 and Additional file 1: Figure S1 shows the representative HRTEM and HAADF-STEM images of two Pt samples. For the Pt-acoustic levitation sample, the single Pt atoms (10%, the numbers of Pt particles with certain size range with respect to total number of Pt particles) coexist with clusters < 1 nm (2% for < 0.5 nm clusters and 25% for 0.5–1.0 nm clusters) and particles larger than 1 nm (46% for 1.0–2.0 nm particles and 17% for > 2 nm particles). In contrast, for the Pt-NaBH_4_ reduction sample, the observation frequency in the range of > 2.0 nm increased sharply (100%) with no single-atom Pt and Pt clusters observed. Figure 1e shows individual Pt atoms dispersed in the Pt-acoustic levitation sample. As shown in Fig. 1b for Pt nanoparticles prepared by the reduction by NaBH_4_ without acoustic levitation, there are no Pt particles with sizes < 2 nm, implying a relatively narrow particle size distribution of large Pt nanoparticles. The success in fabricating single-atom Pt may lie in the extremely weak Pt-Pt interaction due to the acoustic levitation [8].

**Fig. 2:**
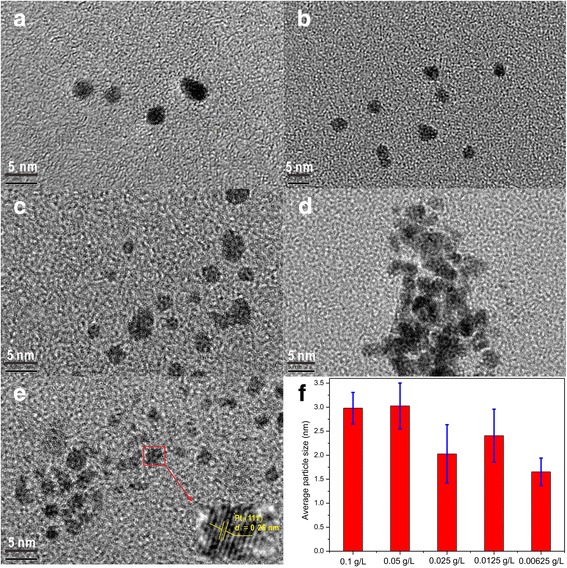
HRTEM images of **a**–**e** Pt nanoparticles prepared by the acoustic levitation method with different metal concentrations (0.1, 0.05, 0.025, 0.0125, and 0.00625 g/L) and **f** average particle size for the Pt clusters

Generally speaking, noble metal nanoclusters with small particle size have high catalytic activity, good light transparency, and obvious size-dependent properties [9–11]. The average particle size of the Pt nanoclusters prepared by the acoustic levitation method is slightly dependent on the concentration of H_2_PtCl_6_ in the starting PVA (polyvinyl alcohol) solution, which is quite different from the usual cases for the chemical reduction preparation of metal colloids [12]. High-resolution TEM images show no aggregates or overgrowth of Pt nanoclusters except for the metal concentration of 0.0125 g L^−1^ (Fig. 2a–e). We have calculated the average diameters of the Pt particles at different metal concentrations based on the certain numbers of Pt particles. For example, Pt particles are uniform with an average diameter of 1.65 ± 0.29 nm (see Fig. 2f and Additional file 1: Figure S2) at the metal concentration of 0.00625 g L^−1^ based on 23 Pt particles in Fig. 2e. The high-resolution TEM image confirms the lattice fringes of Pt (111) planes with *d*-spacing of ~ 0.26 nm.

**Fig. 3:**
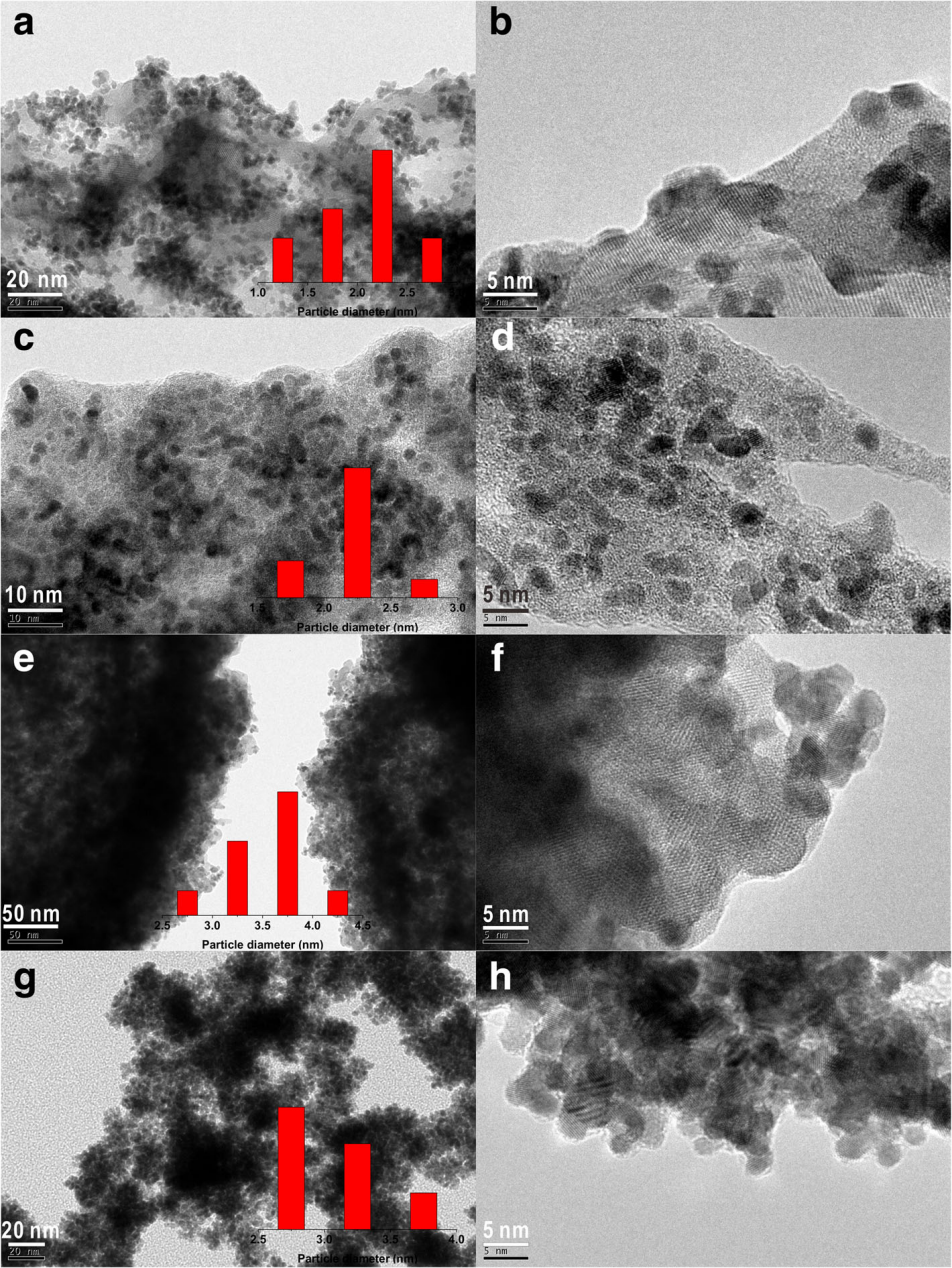
High-resolution TEM images of Pt nanoparticles supported on La_2_O_3_ prepared by the **a**, **b** ultrasonication-acoustic levitation method, **c**, **d** acoustic levitation method, **e**, **f** ultrasonication-colloidal deposition method, and **g**, **h** conventional colloidal deposition method

We also examined the growth of metal clusters by supporting on the surface of rare earth oxide (i.e., La_2_O_3_). HRTEM images taken after Pt ultrasonication-acoustic levitation deposition on La_2_O_3_ at metal concentration of 0.5 g L^−1^ showed that the average Pt particle diameter remained at ~ 2.0 nm (Fig. 3a). It confirmed the presence of essentially monodispersed Pt particles deposited on the surface of the support. As one can see, the Pt particles on the surface of the support are strongly adsorbed on the oxide. Furthermore, the adsorption effect does seem to change the shape of these particles (from spheres to irregular particles) (Fig. 3b). The Pt particles appear to be buried into the oxide support. Our results suggest that the support interacts with Pt clusters, leading to a change in the shape of the Pt particles.

The average diameter for typical acoustic levitation prepared Pt particles without pretreatment of ultrasonication was 2.3 nm (Fig. 3c). Interestingly, the HRTEM image shows that tiny Pt particles are uniformly decorated on the surface of La_2_O_3_ nanosheets without aggregation. All the Pt clusters are anchored on the surface of support, and no Pt nanocrystals are dropped off from the nanosheets even under powerful ultrasound, indicating that the Pt clusters are tightly adsorbed on the surface of La_2_O_3_ support with strong interactions. There are strong differences in the shapes for the Pt particles, and various geometries seem to be possible on the support. This change of geometries could lead to defect formation on the Pt clusters [13].

To check for possible other changes of the Pt clusters supported on the surface of La_2_O_3_ prepared by only ultrasonication pretreatment without acoustic levitation, HRTEM measurement has been performed on the as-prepared Pt/La_2_O_3_ material. As revealed from the HRTEM image of Pt/La_2_O_3_ (Fig. 3e), we found that a large number of Pt particles with an average size of 3.5 nm were dispersed. However, because no acoustic levitation method was used in the synthesis process, metal nanoparticles should be able to interact with each other, leading to their certain aggregations (Fig. 3f).

In contrast, more conventional Pt/La_2_O_3_ system prepared using the colloidal deposition method contains somewhat larger Pt particles with an average diameter of 3.1 nm (Fig. 3g). Compared to the sample prepared on a vessel wall, Pt nanocrystals grown in the ultrasonic levitation system all showed smaller sizes, more irregular shapes, and fewer free-standing Pt particles. Consequently, various adverse effects from the vessel wall can be avoided during crystallization and Pt nanocrystals were able to grow as we expected. Heterogeneous nucleation on the vessel wall was greatly reduced inside the levitated droplet. Further, the acoustic streaming and nonuniformity of the force field could result in fast mass transfer and uncontrolled sample rotation, which can inhibit the crystallization of Pt particles [14]. Also, it was reported that a long-time ultrasonic process inhibited nucleation of crystals [15, 16].

**Fig. 4:**
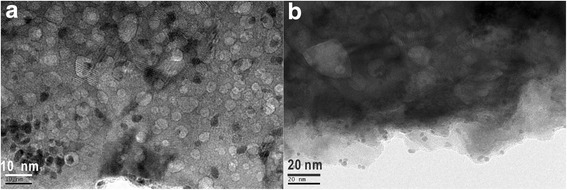
**a**, **b** High-resolution TEM images of Pt nanoparticles supported on La_2_O_3_ prepared by the ultrasonication-acoustic levitation method (metal concentration of 0.00625 g L^−1^)

Generally, different preparation routes for supported metal nanoparticles have been employed, such as physical (e.g., sonication, microwaves, UV (ultraviolet)), chemical (e.g., impregnation, co-precipitation, deposition-precipitation), and physicochemical routes (i.e., sonoelectrochemical) [17]. There are several interesting features in the use of sonication. Ultrasound remarkably enhances mass transport, reduces the diffusion layer thickness, and may also affect the surface morphology of the treated materials [18]. Deposition and reduction of the particles take place almost consecutively. Here, further information on the morphology and structure of the Pt ultrasonication-acoustic levitation deposition on La_2_O_3_ at a metal concentration of 0.00625 g L^−1^ was obtained by electron microscopy (Fig. 4). Interestingly, we found well-distributed and stabilized supported Pt nanoparticles in the well-developed porous La_2_O_3_ support surface (Pt average particle size ~ 2.2 nm). The most attractive feature of the protocol is that the porous materials and the supported noble metal nanoparticles can be produced simultaneously. In general, metal oxides with a specially designed porous structure can be easily functionalized in order to meet the requirements for most applications [19, 20]. By our approach, the synthesis of highly dispersed Pt metal nanoparticles on and/or inside microporous La_2_O_3_ support (i.e., Pt/porous La_2_O_3_) can be one-step realized without any pretreatment/modification of raw oxide.

**Fig. 5:**
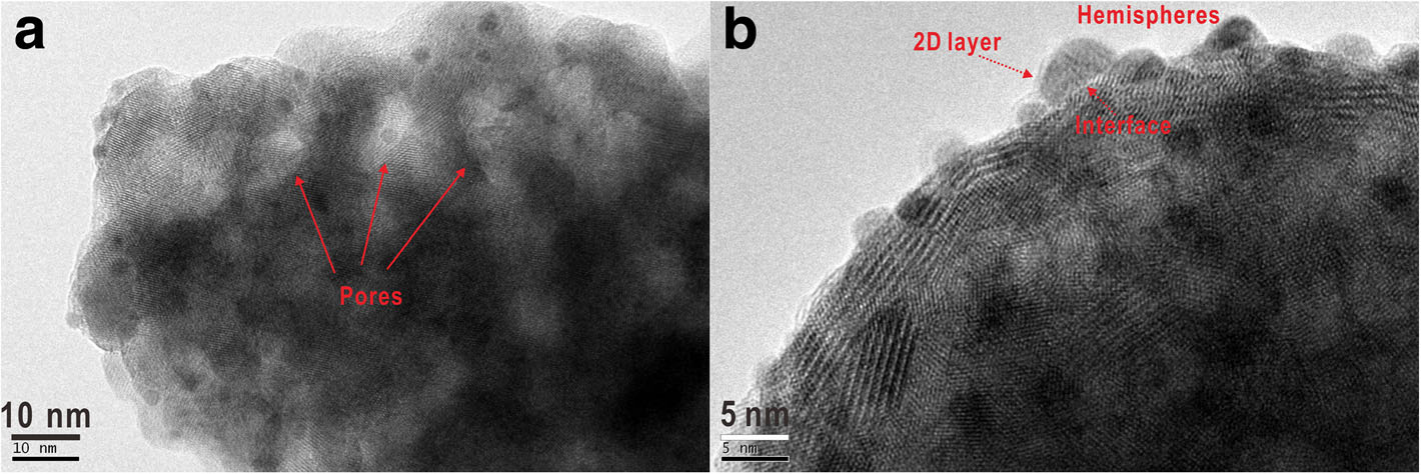
**a**, **b** High-resolution TEM images of Pt nanoparticles supported on La_2_O_3_ prepared by the acoustic levitation method (metal concentration of 0.00625 g L^−1^)

Furthermore, in order to prove the dominant effect of the acoustic levitation on the surface modification of La_2_O_3_, we obtained the morphology information of the Pt acoustic levitation deposition on La_2_O_3_ at a metal concentration of 0.00625 g L^−1^ without pretreatment of ultrasonication method (Fig. 5). From the HRTEM images, we found that the Pt/porous La_2_O_3_ can be still one-step prepared by the simple acoustic levitation method. This result indicates that the acoustic levitation can affect the surface morphology of the La_2_O_3_. This method can be applied to synthesize microporous oxide without any chemical reactions. More interestingly, not only the acoustic levitation can change the morphology and structure of La_2_O_3_, but also there is a strong interaction between Pt nanoparticles and La_2_O_3_ support as shown in Fig. 5b. Acoustic levitation deposition on the oxide does change the shape (i.e., hemisphere) of these Pt nanoparticles in this case. The Pt particles appear to interact with the support materials, and interface between Pt particles and La_2_O_3_ oxide can be distinguished. La_2_O_3_ oxide layer can also cover and contact with the Pt nanoparticles, and this change of geometries (from 3D particle to 2D layer) could lead to defect formation on the Pt particles. These aspects may be of importance because such defects and interfaces can act as active sites on which the catalytic oxide reaction could take place [21, 22]. This finding could strongly contribute to the synthetic methodology of the surface/interfacial heterogeneous catalysts.

**Fig. 6:**
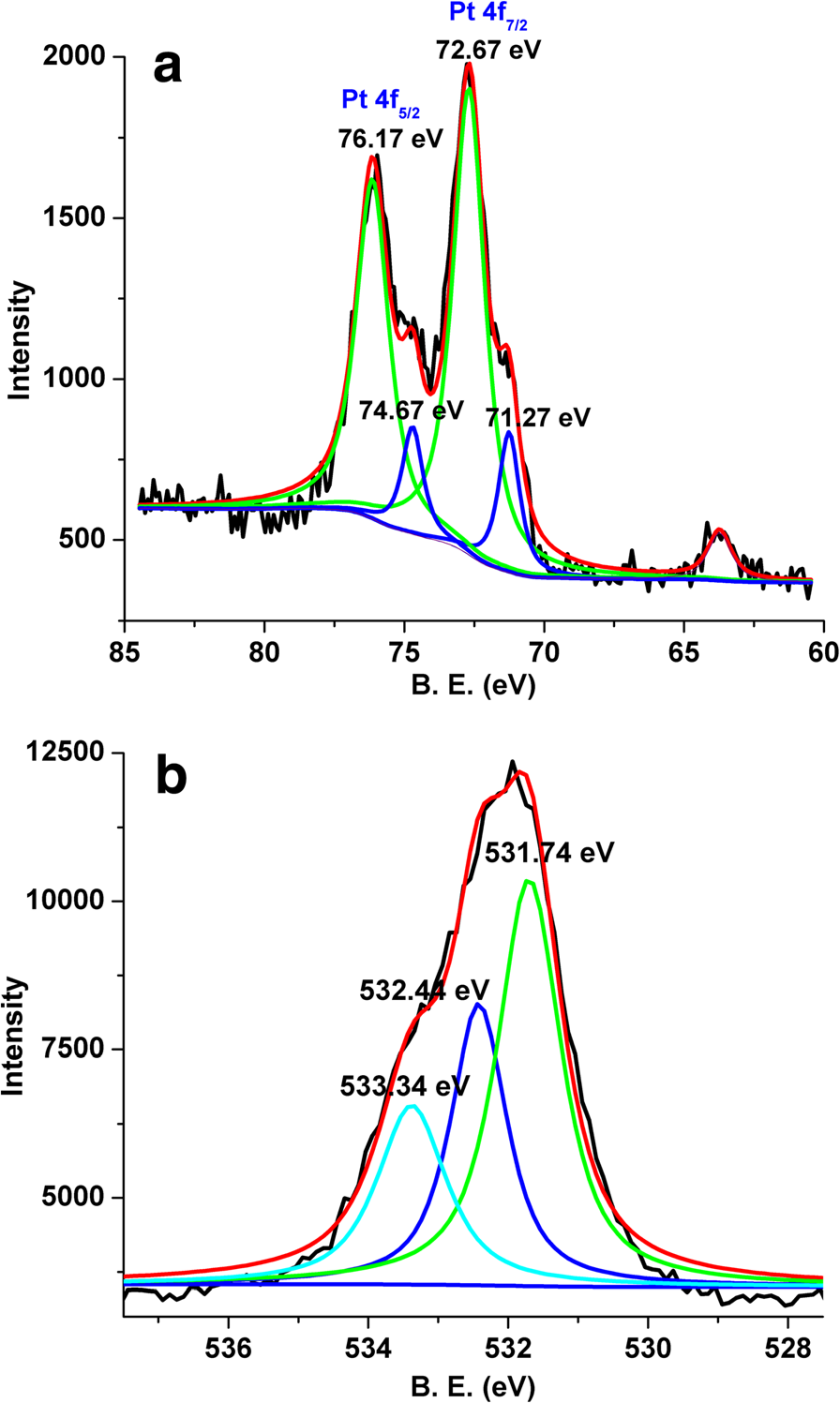
XPS spectra of **a** Pt 4f and **b** O 1s for Pt/La_2_O_3_ sample

Finally, we have done the XPS to confirm the oxidation state of Pt (Fig. 6a). Two Pt states, represented by Pt 4f_7/2_ signals at BE 71.27 and 72.67 eV, can be identified. The first one corresponds to Pt at a zero valent state [23]. The BE position of the second peak can be considered as a result of the formation of Pt–OH bound and oxidized surface compounds (i.e., PtO_*x*_). Based on the above XPS analysis, we can infer that the La_2_O_3_ oxide layer indeed covers and contacts with the Pt metal, leading to the formation of surface La–O–Pt species, which is similar with the result of HRTEM in Fig. 5b. Figure 6b shows the O 1s core-level spectra for the Pt/La_2_O_3_ sample. The O 1s spectrum can be decomposed into three components at B. E. = 531.74, 532.44, and 533.34 eV: the first one is due to the surface lattice oxygen (O_latt_) species, whereas the second one is due to the surface adsorbed oxygen (O_ads_) species, and the last one can be assigned to the electrophilic O-species (O_2_^−^ or O^−^), indicating that the Pt/La_2_O_3_ possesses various oxygen adspecies and may facilitate the electrophilic oxidation reaction [10, 11].

Acoustic levitation technology can simulate the outer space environment in the earth environment. It provides ideal experimental conditions for researching and preparing various high-quality materials and exploring new materials. Acoustic levitation provides a container-free condition, which is helpful to identify the effect of solid wall on materials synthesis. Researches on nanomaterials synthesis under acoustic levitation would obtain deeper insight into the nucleation, aggregation, and dynamics in the systems. In this work, we can conclude that the container-free condition plays an important role in the synthesis of Pt/microporous La_2_O_3_ materials where there is a strong interaction between Pt nanoparticles and La_2_O_3_ support.

## Conclusions

In summary, we have successfully prepared single-atom Pt material in the solution and supported Pt nanoclusters on microporous La_2_O_3_ by a one-step acoustic levitation method without any pretreatment/modification of raw oxide. We found that the acoustic levitation could effectively affect the surface morphology of the La_2_O_3_. Furthermore, the Pt particles appear to interact with the support materials and interface between Pt particles and La_2_O_3_ oxide can be distinguished. La_2_O_3_ oxide layer can also cover and contact with the Pt nanoparticles, and this change of geometries (from 3D particle to 2D layer) could lead to defect formation on the Pt particles.

## Methods

**Fig. 7:**
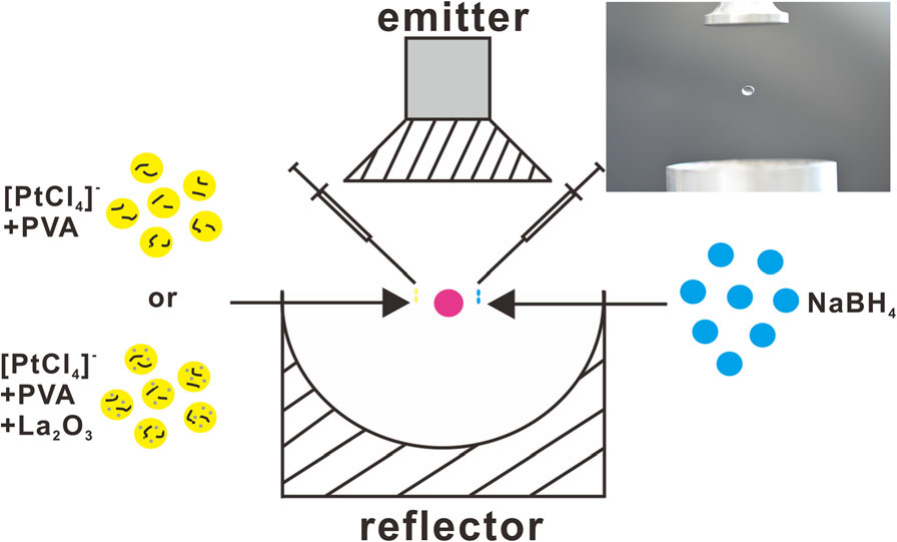
Schematic view of the experimental setup of the acoustic levitator

The acoustic levitator used here is composed of an emitter and a reflector and worked at a fixed frequency of 30 kHz, as shown in Fig. 7. We levitated the liquid sample via the acoustic radiation force exerted on the sample surface as a result of the nonlinear effect of ultrasound [8].

### Preparation of Pt Sol

In a typical preparation, the protecting agent (PVA) was added to an aqueous HPtCl_4_ solution (metal concentration 0.5, 0.1, 0.05, 0.025, 0.0125, and 0.00625 g L^−1^) at room temperature under vigorous stirring. The obtained solution was then levitated via ultrasound for several seconds. A following injection of an aqueous solution of NaBH_4_ (0.005 mol L^−1^) led to the formation of the Pt sol.

### Preparation of Pt/Microporous La_2_O_3_

The La_2_O_3_ support which was synthesized using the d-glucose and lanthanum nitrate (G:M = 1:1.85) by hydrothermal method at 180 °C for 20 h [24] was added to the aqueous HPtCl_4_ solution (metal concentration 0.5 and 0.00625 g L^−1^) under ultrasonic dispersion or not and then levitated via ultrasound for several seconds. A following injection of an aqueous solution of NaBH_4_ (0.005 mol L^−1^) led to the formation of the Pt/microporous La_2_O_3_ material. The Pt loading in Pt/La_2_O_3_ sample prepared by acoustic levitation is 1.01 wt%.

### Materials Characterization: Microscopy experiments

The morphological characterization of the Pt sol and Pt supported microporous La_2_O_3_ material was performed with a JEOL JEM-2100 microscope. Aqueous samples were deposited to a thin film carbon grid and left to dry in the air. Particle sizes and particle size distribution were determined from the TEMs by measuring the sizes of tens of particles. The chemical state of surface element in the Pt/La_2_O_3_ sample was measured by X-ray photoelectron spectroscopy (Perkin-Elmer, ESCA PHI 5400).

## Additional file

Additional file 1: HAADF-STEM and HRTEM images of Pt particles. (DOCX 2196 kb)

## Abbreviations

2D: Two-dimensional
3D: Three-dimensional
HRTEM: High-resolution transmission electron microscopy
PVA: Polyvinyl alcohol
UV: Ultraviolet
XPS: X-ray photoelectron spectroscopy
