# Supplementary material for: Fast Synthesis of Pt Nanocrystals and Pt/Microporous La2O3 Materials Using Acoustic Levitation
Source: Nanoscale Res Lett. 2018 Feb 13;13:50. doi: 10.1186/s11671-018-2467-8 (PMC5809625; doi:10.1186/s11671-018-2467-8)
Supplement: Supplementary file 1 — HAADF-STEM and HRTEM images of Pt particles. (DOCX 2196 kb) [file 11671_2018_2467_MOESM1_ESM.docx]

Fast synthesis of Pt nanocrystals and Pt/microporous La_2_O_3_ materials using acoustic levitation

*Yinkai Yu, Shaohua Qu, Duyang Zang, Liuding Wang and Hongjing Wu **

Key Laboratory of Space Applied Physics and Chemistry of Ministry of Education, Department of Applied Physics, School of Sciences, Northwestern Polytechnical University, Xi’an 710072, P. R. China;

***** Correspondence: wuhongjing@mail.nwpu.edu.cn; Tel.: +86-029-88431664


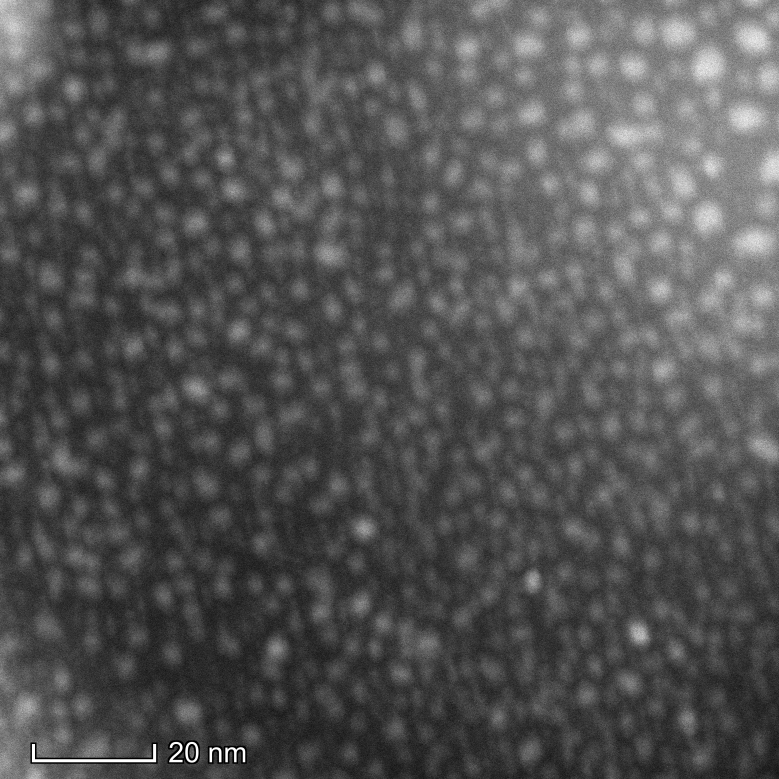


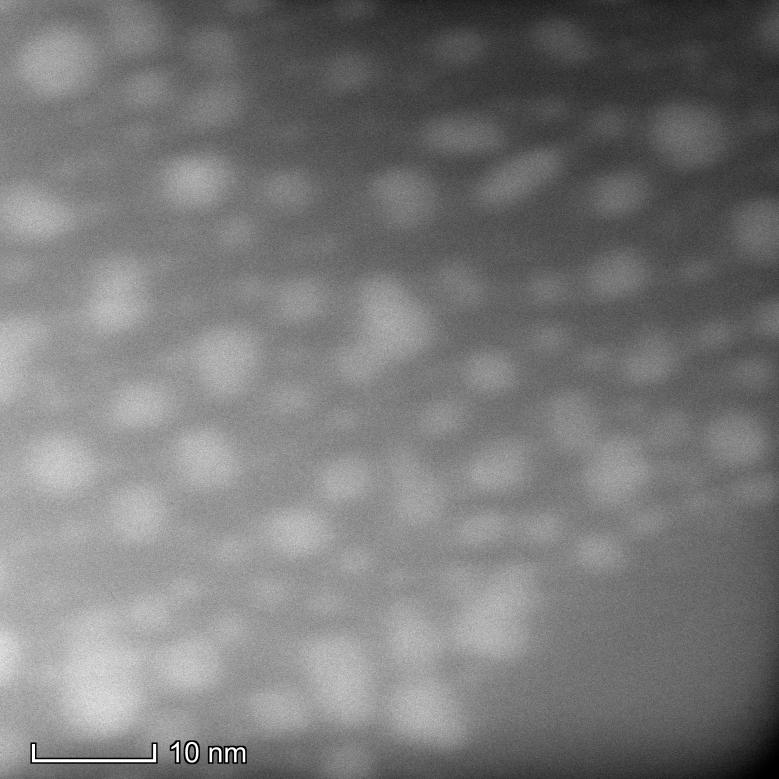


Fig. S1 HAADF-STEM images of Pt clusters prepared by the acoustic levitation (0.5 g L^-1^).


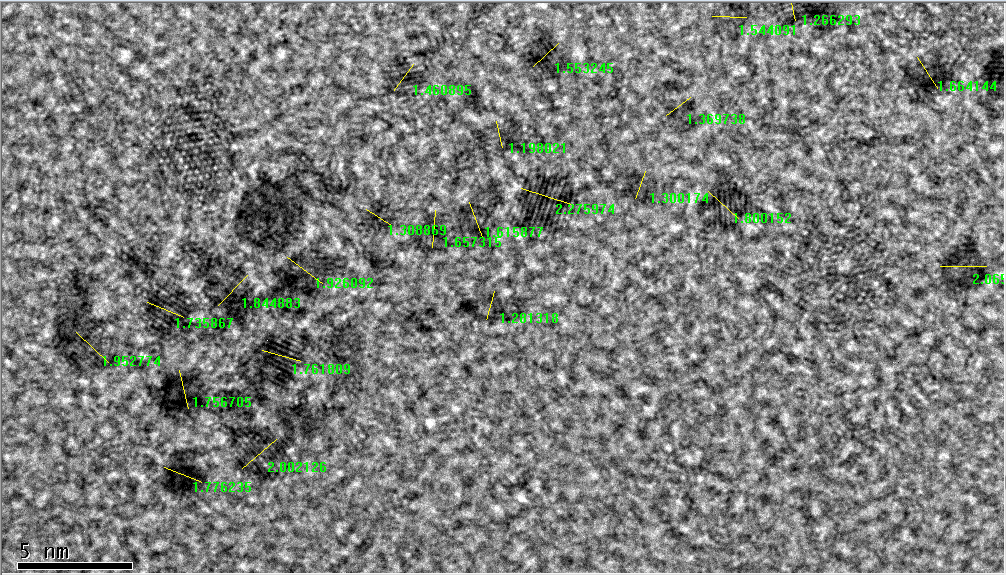


Fig. S2 The average diameter analysis procedure of Pt nanocrystals (0.00625 g L^-1^).
